# Multidisciplinary management in chronic myeloid leukemia improves cardiovascular risk measured by SCORE

**DOI:** 10.3389/fphar.2023.1206893

**Published:** 2023-07-19

**Authors:** Alberto Blanco Sánchez, Rodrigo Gil Manso, Gonzalo Carreño-Tarragona, Diana Paredes Ruiz, Jesús González Olmedo, Joaquín Martínez-López, Carmen Díaz Pedroche, Rosa Ayala

**Affiliations:** ^1^ Department of Hematology, Hospital Universitario 12 de Octubre, Madrid, Spain; ^2^ Department of Medicine, Hospital Universitario 12 de Octubre, Madrid, Spain

**Keywords:** chronic myeloid leukemia (CML), cardiovascular risk, tyrosine kinase inhibitor (TKI), SCORE, multidisciplinary management, coronary disease, thromboembolic disease

## Abstract

**Introduction:** Cardiovascular events are one of the main long-term complications in patients with chronic myeloid leukemia (CML) receiving treatment with tyrosine kinase inhibitors (TKIs). The proper choice of TKI and the adequate management of risk factors may reduce cardiovascular comorbidity in this population.

**Methods:** This study evaluated the cardiovascular risk of a cohort of patients with CML at diagnosis and after follow-up in a specialized cardiovascular risk consultation. In order to do this, we performed data analysis from 35 patients who received TKIs and were referred to the aforementioned consultation between 2015 and 2018 at our center. Cardiovascular risk factors were analyzed separately, as well as integrated into the cardiovascular SCORE, both at diagnosis and at the last visit to the specialized consultation.

**Results:** At the time of diagnosis, 60% had some type of risk factor, 20% had a high or very high risk SCORE, 40% had an intermediate risk, and 40% belonged to the low risk category. During follow-up, the main cardiovascular adverse event observed was hypertension (diagnosed in 8 patients, 23%). 66% of patients quit smoking, achieving control of blood pressure in 95%, diabetes in 50%, weight in 76%, and dyslipidemia in 92%. 5.7% of patients suffered a thrombotic event and a significant percentage of patients showed a reduction in their SCORE.

**Conclusion:** Our study shows the benefit of controlling cardiovascular risk factors through follow-up in a specialized consultation for patients with CML treated with TKI.

## 1 Introduction

The introduction of tyrosine kinase inhibitors (TKIs) in the treatment of chronic myeloid leukemia (CML) marked a significant change in the management and prognosis of this disease (Berman, 2022). This family of drugs allowed higher survival rates of these patients to a level like that of the general population (Hochhaus et al., 2020). Moreover, TKIs helped in achieving symptom control, total clearance of the tumor clone, and significantly reducing the rate of acute transformation (Cortes et al., 2021).

However, TKI treatment poses new challenges in the management of CML, like those associated with the numerous interactions of these drugs and the adverse effects derived from their use (Haouala et al., 2011). Among the latter, the most frequent and concerning are cardiovascular side effects (Douxfils et al., 2016) (Dahlén et al., 2016), which raise the need for strict control of cardiovascular risk factors at the time of diagnosis or those emerging over the follow-up (Barber et al., 2017).

Currently, five TKIs with similar efficacy rates and a different toxicity profile are approved for the treatment of CML (García-Gutiérrez & Hernández-Boluda, 2019). Generally, patients experience some type of (mostly mild) adverse effect, that may sometimes prompt a change in TKI (Cortes & Kantarjian, 2016).

The mechanism by which TKIs cause cardiovascular damage is not fully characterized, although it appears to be related to endothelial damage through non-specific inhibition of tyrosine kinases (“off-target” effect), alteration of glycemic metabolism, direct hypertensive effect or glomerular impairment (Chaar et al., 2018).

There are no studies comparing directly second-generation TKIs (dasatinib, nilotinib, bosutinib), but the results of studies comparing these with imatinib show a higher rate of cardiovascular events with this generation of TKIs, so imatinib may be a preferable option in patients with a high risk of cardiovascular disease (Cortes, 2020).

Furthermore, no clear consensus exists on when to refer a patient with CML from the hematology consultation to another specialist for the evaluation and management of cardiovascular risk. Guidelines on this matter recommend doing so in the case of a history of cardiovascular disease (Seguro et al., 2021), high risk of cardiovascular disease (Zamorano et al., 2016) or presence of risk factors when starting high risk TKI such as nilotinib (NCCN Clinical Practice Guidelines in Oncology, 2023). There are no specific recommendations to this effect from the European Leukemia Net.

However, at the time of diagnosis, patients diagnosed with CML presented a high prevalence of cardiovascular risk factors, which seems to be higher than that of the general population (Seguro et al., 2021). One study showed, at the time of CML diagnosis, a prevalence of 30% of hypertension, 11% of diabetes and 18% of dyslipemia (Coutinho et al., 2017).

Most of our knowledge about the efficacy and adverse effects of TKIs comes from clinical trials. Nevertheless, their results could underestimate the development of cardiovascular comorbidity, considering the exclusion of patients with insufficient control of cardiovascular risk factors, or the younger average age of patients included in the main first-line trials with dasatinib (Kantarjian et al., 2010) or nilotinib (Saglio et al., 2010).

Therefore, real world evidence studies are essential, as they are able to show the prevalence of complications arising from the use of TKIs in a routine clinical practice scenario. One of the largest studies to date (Coutinho et al., 2017), showed a prevalence of almost 80% of cardiovascular risk factors at 5 years after the diagnosis of CML.

In this study, we report our experience in the management of cardiovascular risk factors at our center, where patients are referred to a specialized internal medicine consultation at diagnosis or during follow-up. The purpose of this strategy is to optimize the control of cardiovascular risk factors. Only symptomatic patients are referred to other specialized consultation (cardiology or angiology).

To analyze the impact of this intervention, we have used the SCORE (Systematic Coronary Risk Evaluation) model, which estimates the risk of death from cardiovascular causes in 10 years. It has the advantage of being adjusted to different European countries, and estimates mortality associated with all atherothrombotic manifestations and not just coronary mortality, unlike the Framingham score. Moreover, SCORE is straightforward to calculate because it includes few parameters: age, sex, systolic blood pressure, total cholesterol and smoking (Visseren et al., 2021).

This model has already been used by other researchers to evaluate the risk of developing cardiovascular events in patients with CML treated with different TKIs, demonstrating its predictive value at diagnosis (Breccia et al., 2015; Caocci et al., 2019).

## 2 Materials and methods

### 2.1 Study design

This is a retrospective, single-center observational study that analyzed a total of 35 patients diagnosed with CML at the 12 de Octubre University Hospital, referred to the cardiovascular disease consultation between 2015 and 2018, who received treatment with one of the approved TKIs for this indication (imatinib, dasatinib, nilotinib, bosutinib and ponatinib). The patients received outpatient follow-up in hematology consultation and by an internal medicine specialist in the aforementioned cardiovascular control consultation.

The diagnosis of CML was made following criteria established by the latest classification of hematological neoplasms published by the WHO (Swerdlow et al., 2017). The following prognostic scores for CML were applied to the diagnosis: Sokal, Hasford, EUTOS and ELTS. Regarding the criteria used to define cardiovascular risk factors, they are explained below.

### 2.2 Cardiovascular variables

Arterial hypertension: defined as systolic blood pressure ≥140 mmHg and/or diastolic blood pressure ≥90 mmHg, following the criteria used by the ESC/ESH Guidelines for the management of arterial hypertension (Williams et al., 2018). Arterial hypertension was considered to be controlled according to the target for general and specific subgroups of hypertensive patients, following the mentioned guidelines.

Dyslipidemia: defined as hypertriglyceridemia (triglycerides level >200 mg/dL) and/or hypercholesterolemia (cholesterol level >200 mg/dL), following the criteria from the ESC/EAS Guidelines for the management of dyslipidaemias (Mach et al., 2020). Dyslipidemia was considered to be controlled following the criteria defined by these guidelines.

Diabetes mellitus: defined as an A1C ≥ 6.5%; fasting blood glucose ≥126 mg/dL; blood glucose ≥200 mg/dL 2 hours after a 75 mg intake of glucose; or a casual blood glucose ≥200 mg/dL, according to the ESC Guidelines on diabetes (Cosentino et al., 2020). Control of diabetes was defined according to the targets specified by these guidelines.

Alcohol abuse: defined following criteria from DSM-V (APA, 2013).

SCORE (Systematic Coronary Risk Evaluation): defined following ESC criteria (Visseren et al., 2021).

### 2.3 Statistics

Frequencies were calculated as percentages for qualitative variables and as means and standard deviations for quantitative variables. Comparison of variables was carried out using the McNemar-Broker test. A *p* < 0.05 was considered statistically significant. Statistical analysis was conducted using the SPSS computer program version 25.0 (IBM, Chicago, IL).

## 3 Results

Table 1 summarizes the main characteristics of the 35 patients included in the study. The mean age at the time of referral to the cardiovascular control consultation was 50 years (standard deviation, 13.5). 45.7% of patients were women. Over half of the patients (51.4%) were classified in the low risk category according to the Sokal index, 60% according to the Hasford score, and 60% according to the ELTS score. However, most patients belonged to the high risk group according to the EUTOS Score (77.1%).

**TABLE 1:** Baseline characteristics of patients (*n* = 35).

*Gender*	
*Male, n (%)*	19 (54.3%)
*Female, n (%)*	16 (45.7%)
*Median age at diagnosis* (*SD*)	50.51 years (13.57)
*Sokal Index*	
*Low, n (%)*	18 (51.4%)
*Intermediate, n (%)*	9 (25.7%)
*High, n (%)*	8 (22.9%)
*Hasford Score*	
*Low, n (%)*	21 (60%)
*Intermediate, n (%)*	10 (28.6%)
*High, n (%)*	2 (5.7%)
*Unknown, n (%)*	2 (5.7%)
*EUTOS Score*	
*Low, n (%)*	6 (17.1%)
*High, n (%)*	27 (77%)
*Unknown, n (%)*	2 (5.7%)
*ELTS Score*	
*Low, n (%)*	21 (60%)
*High, n (%)*	12 (34.3%)
*Unknown, n (%)*	2 (5.7%)

Regarding the prescribed TKI (Table 2), all except 2 patients received imatinib (median time of exposition, 20.3 months), 45.7% received dasatinib (median time of exposition, 24 months), 42.9% received nilotinib (median time of exposition, 15.5 months), 3 patients received bosutinib (median time of exposition 4.1 months), and 1 received ponatinib (7.3 months of exposition). 60.6% of patients treated with imatinib had to stop it (in 40% of these cases due to lack of optimal response, 35% as a result of adverse effects, and 25% because of clinical trial protocol). Patients who stopped dasatinib (57.7% of those who received this drug) did so for reasons related to adverse effects. The discontinuation rate with nilotinib was 60% (in one because of lack of efficacy and in the remaining 88.9% as a consequence of toxicity). Out of the three patients treated with bosutinib, one stopped it due to toxicity, and the only patient treated with ponatinib stopped it because of clinical trial protocol.

**TABLE 2:** TKI (tyrosine kinase inhibitor) received for CML.

Imatinib, n (%)	33 (94.3%)
Suspension	20 (60.6%)
Reasons	
Lack of response	8 (40%)
Toxicity	7 (35%)
Clinical Trial	5 (25%)
Dasatinib, n (%)	16 (45.7%)
Suspension	8 (50%)
Reason	AE: 8 (100%)
Nilotinib, n (%)	15 (42.86%)
Suspension	9 (60%)
Reasons	
Lack of response	1 (11.11%)
Toxicity	8 (88.89%)
Bosutinib, n (%)	3 (8.6%)
Suspension	1 (33.34%)
Reason: Toxicity	1 (100%)
Ponatinib, n (%)	1 (2.9%)
Suspension	1 (100%)
Reason: Clinical Trial	1 (100%)
TKI, total number used during follow-up, n (%)	
1	15 (42.8%)
2	10 (28.6%)
3	9 (25.7%)
4	1 (2.9%)

Table 3 shows the proportion of patients who had some type of cardiovascular risk factor either at the time of referral or at the last visit to the cardiovascular control consultation. The time elapsed between diagnosis and consultation was approximately 54 months on average. At the time of the consultation 17.1% had an active tobacco habit and 28.6% had stopped smoking. 11.4% had alcohol consumption in the range of abuse according to the previously stated criteria. 34.3% had hypertension, 8.6% had DM, and 40% had dyslipidemia. Seven patients had already developed cardiovascular disease at the time of the consultation (2 in the form of coronary disease, 2 stroke, and 3 peripheral arterial obstructive disease). One patient had a diagnosis of chronic obstructive pulmonary disease COPD and 2 had chronic kidney disease.

**TABLE 3:** Cardiovascular Risk Factors at first visit and last follow-up in Specialized Consultation.

*Months from diagnosis to referral, mean (SD)*	53.80 (75.1)	
*Smoking, n* (*%*)	At first visit	At last follow-up
Active	6 (17.14%)	2 (5.71%)
Previous	10 (28.57%)	14 (40.00%)
No smoking	19 (54.29%)	19 (54.29%)
*Alcohol abuse, n* (*%*)	4 (11.4%)	2 (5.7%)
*Arterial Hypertension, n* (*%*)	12 (34.3%)	20 (57.14%)
Improvement in blood pressure levels		20 (100%)
Control of hypertension		19 (95%)
*Diabetes mellitus type 2, n* (*%*)	3 (8.6%)	6 (17.2%)
Improvement in glucose levels		6 (100%)
Control of diabetes		3 (50%)
*Dyslipidemia, n* (*%*)	14 (40%)	24 (68.57%)
Improvement in lipid levels		24 (100%)
Control of dyslipidemia		22 (91.66%)
*Cardiovascular Disease, n* (*%*)	7 (20.00%)	11 (31.4%)
*Coronary Disease*	2 (5.7%)	3 (8.57%)
*Cerebrovascular Disease*	2 (5.7%)	2 (5.7%)
*Peripheral Arterial Disease*	3 (8.6%)	6 (17.2%)
*COPD*	1 (2.90%)	1 (2.9%)
*Chronic kidney disease*	2 (5.70%)	2 (5.7%)
SD: standard deviation; COPD: chronic obstructive pulmonary disease		

During a mean follow-up of 31.25 months, 3 patients were diagnosed with diabetes, 8 developed hypertension (13.3% of patients with nilotinib, 12.5% with dasatinib and 12.1% with imatinib), 10 dyslipidemia and 3 peripheral arterial obstructive disease (PAOD). Strict control of hypertension was achieved in all but one patient, control of dyslipidemia in all but 2 and only 3 patients did not reach adequate diabetes control. However, there was an improvement of blood pressure, glucose level and lipids in all patients. In 12 patients, it was necessary to change either the type or dosage of TKI because of interactions with concomitant medication, with statins being the main reason in 75% of these cases.

The most frequent cardiovascular disease in our cohort was PAOD (6 patients developed PAOD after CML diagnosis, 3 of them before referral to Internal Medicine Department and 3 of them afterwards). The median age at the time of PAOD diagnosis was 63.5 years, with a median time of 13.15 years from the introduction of TKI treatment. Regarding the former 3 patients, 2 were receiving nilotinib and 1 dasatinib. Two of them belonged to the intermediate risk and 1 to the very high risk SCORE category. An improvement of SCORE was reached in 2 of them.

The latter 3 cases with PAOD were diagnosed with a median of 2.5 years after the first consultation. All of them were receiving imatinib (with a median time of exposition of 13.7 years). One of them belonged to the high risk group and 2 to the intermediate risk group. All of them remained in the same SCORE category, despite the adequate control of cardiovascular risk factors.

**FIGURE 1:**
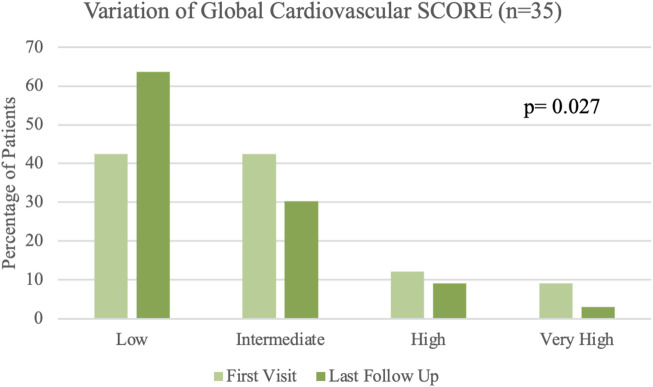
SCORE at the time of the first visit and at last follow up.

The Figure 1 shows the distribution of patients according to the cardiovascular SCORE at the time of the first consultation and the last one. We have observed an increased number of patients belonging to the low risk group at the expense of a decrease in those assigned to the intermediate, high, and very high risk groups, with a difference that reaches statistical significance.

The distribution of the patients among the different groups before and after follow-up is low risk (21 *versus* 14 patients), intermediate risk (10 *versus* 14) and high risk (3 *versus* 2). Only one out of three patients remained in the very high risk category.

Regarding arterial thrombosis, one patient receiving treatment with dasatinib presented an episode of acute coronary syndrome. He had a history of hypertension, dyslipidemia and coronary disease prior to TKI initiation, belonging to the high risk SCORE category when starting follow-up.

With respect to the data on thromboembolic disease, only one patient (receiving imatinib as TKI) presented a venous thrombotic event in the form of deep vein thrombosis in the lower limb, arising in the postoperative context of a major abdominal surgery. A patient with a history of antiphospholipid syndrome and deep vein thrombosis prior to the diagnosis of CML received imatinib without new thrombotic events after the start of this drug. No patient treated with second generation TKI or ponatinib developed venous thrombotic events.

At the end of follow-up, 8 patients (22.9%) had been referred to the vascular surgery and angiology consultation. Out of the 8 patients, 7 were referred because of intermittent claudication and 1 for multidisciplinary assessment due to very high cardiovascular risk.

These patients were evaluated with lower limb and carotid doppler. Half of them showed carotid atherosclerosis, but only one presented with significative stenosis (more than 50% of arterial diameter reduction).

Ten patients underwent lower limb doppler in order to rule out significant arterial obstruction. Three patients showed findings of arterial obstruction (those diagnosed with PAOD), four atherosclerotic plaques and three did not reveal pathologic findings.

## 4 Discussion

In this paper we present the results of cardiovascular control in patients with CML under treatment with TKI in a specific consultation. A reduction in cardiovascular risk factors was achieved with at least a 20% improvement in cardiovascular score.

The baseline characteristics of our cohort are similar to those reported previously in patients with CML: an average age of 57 years and a slight predominance in males (Dahlén et al., 2016). As for the cardiovascular risk factors in our series, the data are comparable to those reported by other authors. The study by Coutinho et al. (Coutinho et al., 2017) showed a rate of hypertension of approximately 30%, like that of our population, and 11% of diabetes (in our study 8.6%). The high proportion of patients with dyslipidemia (40% compared to 18% in the aforementioned study) in our cohort is striking, a difference that may be due to heterogeneity of criteria used to define this condition.

The presence of cardiovascular risk factors or comorbidities is important, on the one hand, for the choice of TKI, given the different toxicity profile of each one, and on the other hand, for the management of such comorbidity (Latagliata et al., 2021). Thus, given that most of our patients received treatment with imatinib and we have a small proportion of patients who received new generation TKIs, it is difficult to make inferences about the relative risk for the development of cardiovascular comorbidity regarding the TKI.

However, according to previous studies, it seems that nilotinib is more associated with the development or worsening of arterial hypertension (Roa-Chamorro et al., 2021), as well as coronary disease together with dasatinib (Barber et al., 2017). Nilotinib is especially associated with stroke (Chen et al., 2021), as well as peripheral arterial disease together with dasatinib (Chen et al., 2021). However, treatment with ponatinib has been the most associated with hypertension (17% vs. 10%) for all new-generation TKI in a pooled analysis of hypertension incidence (Mulas et al., 2021) and thrombotic risk (10% patients developed cerebrovascular or vaso-occlusive disease) (Jain et al., 2015).

For this reason, patient-based therapy has become increasingly important in the treatment of CML (Ciftciler & Haznedaroglu, 2021). The availability of several TKIs has made it possible to choose the most appropriate drug for each patient based on individual factors such as age, comorbidities and availability in each center (Rabian et al., 2019). It is important to consider factors such as the patient’s overall health status, potential side effects, and the risk of developing resistance to the TKI when selecting the best option. In summary, a personalized approach to CML treatment can improve outcomes by maximizing the benefits of therapy while minimizing side effects and reducing risk of treatment resistance (Ciftciler & Haznedaroglu, 2021).

Given that most of our patients received treatment with imatinib in first line and we have a small proportion of patients who received new generation TKIs, it is difficult to make inferences about the relative risk for the development of cardiovascular comorbidity according to the TKI in our CML cohort. Nevertheless, with a median follow-up of 27.8 months, none of the patients who received second generation TKI and who had previous arterial hypertension showed a worsening of this condition (only a patient with imatinib had a deficient control of hypertension during follow-up). 23% of patients were diagnosed of hypertension at some point after TKI initiation. This percentage is slightly lower than that showed by the large cohort of Jain et al. (2019). The only patient who received ponatinib was under antihypertensive treatment before diagnosis of CML and showed an adequate control of hypertension during TKI therapy.

Although the associated thrombotic risk is assessed as a class effect of TKI, the difference in targets of each of the different TKI may explain the differences observed. The Swedish registry showed that patients with CML have an overall risk of venous thromboembolic events and arterial thromboembolic events 1.5 and 2 times higher than general population, respectively (Dahlén et al., 2016). Moreover, second generation TKI and ponatinib seem to confer greater risk than imatinib (Douxfils et al., 2016). In our cohort, the rate of thromboembolic events was low, and these only occurred in patients with strong risk factors. The comparison with other studies is difficult due to difference of median follow-up (Jain et al., 2019). However, these data suggest that follow-up in the specialized consultation may have been effective in preventing thrombotic events.

PAOD rate was surprisingly high in comparison to other cardiovascular events. Other studies show a greater percentage of coronary or cerebrovascular events, with an incidence lower than 1% of PAOD among patients treated with imatinib (Chen et al., 2021). Half of our patients were receiving imatinib at the time of PAOD diagnosis, although nilotinib seems to be more associated with PAOD than other TKI (Douxfils et al., 2016). Yet our patients had a long history of exposition and many cardiovascular risk factors. Our high rate of PAOD could be a consequence of the high suspicion degree maintained in the specific consultation. There are many comorbidities causing lower limbs pain and, unlike cerebrovascular or coronary disease, PAOD is often mildly symptomatic and thus misdiagnosed (Nordanstig et al., 2023). For this reason, nearly one-third of patients underwent a Doppler study and were referred to vascular surgery and angiology consultation.

Another important aspect to consider when controlling cardiovascular risk factors through pharmacological measures is the potential interactions of the TKIs. In our cohort, this had a fundamental impact on the use of statins, as previously seen (Haouala et al., 2011), and for which rosuvastatin or pravastatin are usually recommended, as they are not substrates of CYP3A4 (Osorio et al., 2018).

An appropriate approach to estimating the risk of developing cardiovascular events are prognostic scores, such as the Framingham score, the Pooled Cohort Equations score or the SCORE. Among them, the SCORE model shows many advantages: there are many country-specific versions derived from local data, it is easy to calculate, and it is capable of predicting mortality derived from myocardial infarction, stroke or heart failure over the next 10 years (Caocci et al., 2019).

As results have shown, a significant percentage of patients achieved a change in their risk stratification according to the SCORE, in all cases achieving a better prognosis category than before follow-up, which was achieved thanks to the control of blood pressure, dyslipidemia, or smoking cessation.

Two studies have shown a correlation between the SCORE and the occurrence of cardiovascular events in patients with CML and TKI treatment (although both only included patients with ponatinib) (Breccia et al., 2015; Caocci et al., 2019). Both showed a higher incidence of cardiovascular events in the high and very high risk groups, with a significant difference. In the study by Breccia et al., none of the patients with a low risk SCORE developed cardiovascular disease.

The importance of preventing cardiovascular disease lies in the fact that it is the second leading cause of mortality in cancer patients (Sulpher et al., 2015). For this reason, the importance of a multidisciplinary management of patients with malignant hematological disorders is increasingly been recognized, although we do not find in the literature studies on multidisciplinary management of cardiovascular risk in patients with CML, even when various groups have called attention to this need (García-Gutiérrez et al., 2016; Basile et al., 2022).

Our study shows that this approach to CML patients, in coordination with specialists is feasible and results in an improved control of cardiovascular risk factors. The main limitation of our study is its retrospective nature and the limited number of patients analyzed. In addition, there has been no prolonged follow-up of patients that could demonstrate a reduction in cardiovascular events in patients with a better prognosis SCORE. Among the strengths of the study, it includes patients treated with different TKIs, and the use of a standardized and population-targeted cardiovascular risk model.

## 5 Conclusion

The adverse effects of tyrosine kinase inhibitors are one of the main concerns when treating patients with chronic myeloid leukemia. These are usually related to their off-target effects and each TKI has a different toxicity profile. Cardiovascular events are among their most frequent and life-threatening complications, and their occurrence can influence the choice or switch of TKI. The development of these events can be prevented by controlling risk factors, which often requires an interdisciplinary management. Our study shows that follow-up in a specialized consultation is an attainable feasible approach that can reduce cardiovascular risk of these patients.

## Data Availability

The raw data supporting the conclusion of this article will be made available by the authors, without undue reservation.
